# The Mechanism of Lung and Intestinal Injury in Acute Pancreatitis: A Review

**DOI:** 10.3389/fmed.2022.904078

**Published:** 2022-07-07

**Authors:** Dongling Liu, Linlin Wen, Zhandong Wang, Yang Hai, Dan Yang, Yanying Zhang, Min Bai, Bing Song, Yongfeng Wang

**Affiliations:** ^1^School of Pharmacy, Gansu University of Chinese Medicine, Lanzhou, China; ^2^School of Traditional Chinese and Western Medicine, Gansu University of Chinese Medicine, Lanzhou, China; ^3^County People’s Hospital, Pingliang, China; ^4^Gansu University of Chinese Medicine/Scientific Research and Experimental Center, Lanzhou, China; ^5^Gansu Provincial Engineering Laboratory for Research and Promotion of Quality Standardization of Authentic Medicinal Materials in Gansu Province/Provincial Key Laboratory of Pharmaceutical Chemistry and Quality Research in Colleges and Universities in Gansu Province/Gansu Provincial Laboratory Animal Industry Technology Center, Lanzhou, China

**Keywords:** acute pancreatitis, lung injury, intestinal injury, inflammation response, oxidative stress, endocrine disorders

## Abstract

Acute pancreatitis (AP), as a common cause of clinical acute abdomen, often leads to multi-organ damage. In the process of severe AP, the lungs and intestines are the most easily affected organs aside the pancreas. These organ damages occur in succession. Notably, lung and intestinal injuries are closely linked. Damage to ML, which transports immune cells, intestinal fluid, chyle, and toxic components (including toxins, trypsin, and activated cytokines to the systemic circulation in AP) may be connected to AP. This process can lead to the pathological changes of hyperosmotic edema of the lung, an increase in alveolar fluid level, destruction of the intestinal mucosal structure, and impairment of intestinal mucosal permeability. The underlying mechanisms of the correlation between lung and intestinal injuries are inflammatory response, oxidative stress, and endocrine hormone secretion disorders. The main signaling pathways of lung and intestinal injuries are TNF-α, HMGB1-mediated inflammation amplification effect of NF-κB signal pathway, Nrf2/ARE oxidative stress response signaling pathway, and IL-6-mediated JAK2/STAT3 signaling pathway. These pathways exert anti-inflammatory response and anti-oxidative stress, inhibit cell proliferation, and promote apoptosis. The interaction is consistent with the traditional Chinese medicine theory of *the lung being connected with the large intestine* (fei yu da chang xiang biao li in Chinese). This review sought to explore intersecting mechanisms of lung and intestinal injuries in AP to develop new treatment strategies.

## Introduction

Acute pancreatitis (AP) is one of the common causes of acute abdomen in clinical practice (1, 2). It is closely caused by two predisposing factors – gallstones and alcohol misuse (3). These two factors stimulate the pancreas to secrete a large amount of digestive enzymes, leading to autodigestion, inflammation, edema, and bleeding, and even necrosis of the pancreas or surrounding fatty tissue. Some studies have demonstrated that AP is associated with fat necrosis around the peripancreatic and intra-pancreatic areas. The necrosis develops because adipocytes in the peritoneum are destroyed and digested by inappropriately activated pancreatic enzymes (4). Stored triglycerides in the adipocytes are released and split by pancreatic lipases into fatty acids and glycerol molecules, and the unsaturated free fatty acids increase damage to the surrounding tissues, and systemic inflammation develops (5). Necrosis of adipocytes serves as an important inflammatory source by the release of a huge number of inflammatory mediators (6). In AP, various inflammatory factors trigger the waterfall cascade of inflammatory mediators through the “trigger-like action.” Subsequently, which causes the systemic inflammatory response syndrome (SIRS), and eventually leads to multiple organ dysfunction syndrome (MODS), both of which contribute to the high mortality rate of AP (7).

The lungs and intestine are generally considered to be the most directly targeted organs of AP, and resulting in lung injury and intestinal injury are the main causes of early death in AP patients, with a mortality rate of 60% (8, 9). In AP, intestinal injury is an early event involving damage of intestinal mucosa barrier and altered intestinal mucosal permeability that occurs in AP (10). This injury increase intestinal permeability and cause massive cell death in the intestinal epithelium and basement membrane, leading to the activation of the local response and inducing bacterial translocation out of the gastrointestinal tract. These phenomena contribute substantially to MODS and systemic inflammatory response, being able to damage distant organs (11–13). Of these multiple organs, the lung appears to be the earliest remote organ affected by AP (14). Acute lung injury (ALI) is the dominant death-associated factor in patients who have diffuse alveolar damage on chest X-ray with early-stage AP (8). The generation of inflammatory mediators, including cytokines and chemokines, as well as reactive oxygen species (ROS), is a fundamental cause of AP-induced lung injury. These mediators contribute to the accumulation of macrophages and neutrophils and, later, trigger a cascade of pathological changes in pulmonary microcirculation of the lungs, leading to the occurrence and aggravation of AP-induced lung injury (15, 16). Noteworthy, lung injury is one of the most important components of the MODS triggered by intestinal injury (14). Both lung and intestinal injury can together worsen the severity of AP, eventually resulting in death. Interestingly, it is consistent with the traditional Chinese medicine theory of *the lung being connected with the large intestine* (17).

There are some close connections between the physiology of lungs and intestine and also between the pathological changes and molecular mechanism of intestinal and lung injury caused by AP. Thus, it is necessary to investigate a common intervention strategy for pulmonary and intestinal injuries. This review summarized the AP-related lung and intestinal injury and their mechanisms, and subsequently discussed the intersections of these mechanisms. This review also aimed to explore the underlying co-mechanisms of multiorgan AP injury and find the novel clinical treatment methods as well as provide the valuable insights into clinical practice.

## The Mechanism of Acute Pancreatitis

Acute pancreatitis is a relatively common pancreatic inflammatory disorder, leading to local and systemic inflammation. The main pathological mechanism of AP is activated trypsin-induced self-digestive acute inflammatory response in the pancreas (18, 19). Once pancreatic acinar cells are damaged, acinar cells would release many pro-inflammatory mediators, such as cytokines and chemokines, and stimulate the recruitment and activation of immune cells, including innate immune cells (neutrophils, macrophages, dendritic cells, mast cells, and NK cells), and adaptive immune cells (T cells and B cells), which will consequently exacerbate the pancreatic injury, resulting in necrosis of the pancreas (18, 20, 21). The necrotized pancreatic acinar cells release various kinds of damage-associated molecular patterns (DAMPs), such as high-mobility group box protein 1 (HMGB1), and activate infiltrating immune-associated cells to generate more inflammatory mediators, which in turn accelerate more immune cell infiltration and aggravate inflammation, even contributing to systemic inflammation (20, 21).

Numerous studies have shown that acinar cell necrosis and inflammation are induced by oxidative stress, Ca^2+^ overload, a disorder of organelles, early activation of trypsin, activation of Toll-like receptors 4 (TLR4), nuclear factor kappa B (NF-κB), NOD-like receptor protein 3 (NLRP3), signal transducer and activator of transcription 3 (STAT3), activator protein-1 (AP-1), and mitogen-activated protein kinases (MAPK) signaling pathways, and accumulation of inflammatory cells (16, 22). Extensive studies have suggested that Ca^2+^ overload and oxidative stress are early events in AP. Toxic by-products of AP (such as bile acids or alcohol metabolites) promote the production of ROS and the release of Ca^2+^, affecting pancreatic cell structure and function (23). ROS can also increase the activation of inflammatory signals, such as AP-1, STAT3, and MAPKs (24).

Acute pancreatitis-related multiorgan injury by activating STAT3 pathway. HMGB1, released in stress, promotes pancreatic injury and pro-inflammatory cytokine release and induces the Janus kinase 2 (JAK2)/STAT3 signal pathway, further intensifying the inflammatory reaction (25). TLRs, especially TLR4, are the first factors to become activated. Activated TLR4 and the NF-κB pathway promote cell synthesis, secreting pro-inflammatory cytokines, such as tumor necrosis factor-alpha (TNF-α), interleukin-1β (IL-1β), and IL-6, which can also stimulate NF-κB, leading to its further activation, thus, resulting in persistent and intensified inflammation (26). TLR9 is expressed in resident immune cells of the pancreas, which are predominantly represented by macrophages, and it is an important DAMP receptors upstream of inflammasome activation, and caspase-1, and NLRP3 inflammasome are required for the development of inflammation in AP (20, 27). A recent study demonstrated that NLRP3 inflammasome and gasdermin D (GSDMD) activation-mediated pyroptosis in acinar cells is closely linked to pancreatic necrosis and systemic inflammation in AP (28). Another study recently indicated a new mechanism in which the p53-apoptosis-inducing factor (AIFM2) pathway regulates AP with multiorgan injury via activating transcription factor 6 (ATF6)-mediated apoptosis, suggesting that endoplasmic reticulum (ER)-mitochondrial-nuclear crosstalk plays an important role in AP development (29). Additionally, the inflammatory process is amplified following further secretion and overexpression of adhesion molecules including intercellular adhesion molecule 1 (ICAM-1) and vascular adhesion molecule 1 (VCAM-1), which represent ligands for lymphocyte function-associated antigen 1 on leukocytes and lymphocytes, αLβ2 and CD11a-CD18 on monocytes and integrin macrophage 1 antigen (Mac-1) on neutrophils, while their secretion is promoted by ROS generation and TNF-α itself (30). The potential targeted pathways in AP are shown in Table 1.

**TABLE 1:** Targeted pathways in AP.

Targeted pathways	Influence	Function	References
Nrf2 pathway	up-regulation	antioxidant	(31)
NF-κB, P38MAPK pathways	up-regulation	Inflammation	(26)
ATF6/p53/AIFM2 pathway	up-regulation	Apoptosis and injury	(29)
TLR signaling pathway	up-regulation	Inflammation; apoptosis	(26, 27)
NLRP3 inflammasome pathway	up-regulation	Inflammation; Pyroptosis	(20, 27, 28)
VCAM-1 and the E-selectin regulation pathway	up-regulation	oxidative stress	(30)
STAT pathway	up-regulation	inflammation	(32, 33)

## Mechanisms of Lung Injury in Acute Pancreatitis

**FIGURE 1:**
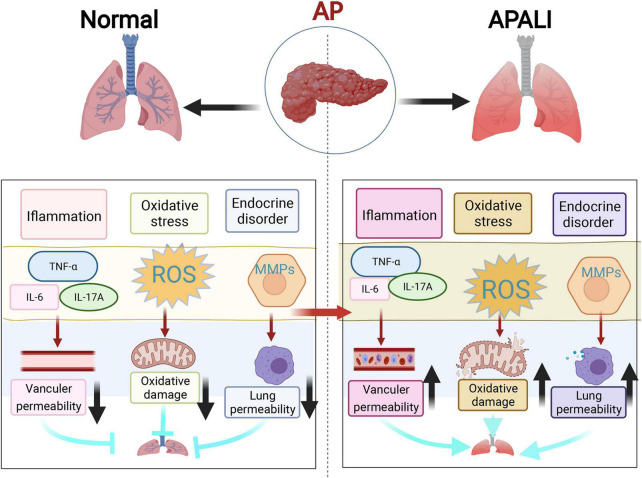
The three main mechanisms of lung injury in acute pancreatitis (AP). This figure shows three most highlighted mechanisms in the studies of lung injury in AP, which include inflammatory response outbreak, oxidative stress damage, and endocrine disorders represented by matrix metalloproteinases (MMPs).

Lung injury is the most common feature and the main cause of early death in AP (8). The pathological changes are closely related to an increase in vascular endothelial cell space and permeability, aggregation of marginal concentration of leukocytes, and profuse expression of ICAM-1 (15, 16). During AP, the accumulation of inflammatory cells and inflammatory mediators in the lung destroys the blood-air barrier. Several studies have demonstrated that that the accumulation of a lot of neutrophils in the lungs enhanced generation of ROS and increased the production of pro-inflammatory cytokines, including HMGB1, endotoxin, TNF-α, IL-1β, and IL-6, which activate various intracellular signaling pathways (e.g., NF-κB, NLRP3, STAT3, MAPK, and AP-1) and adhesion molecules, thus, releasing inflammatory mediators, which exacerbate the damage and result in lung injury (16, 34). According to the relevant literature, the pathological reactions and their molecular mechanisms of AP-related lung injury can be summarized by mainly including inflammatory response outbreak, oxidative stress damage, and endocrine disorders (Figure 1).

### Inflammatory Response of Lung Injury in Acute Pancreatitis

**FIGURE 2:**
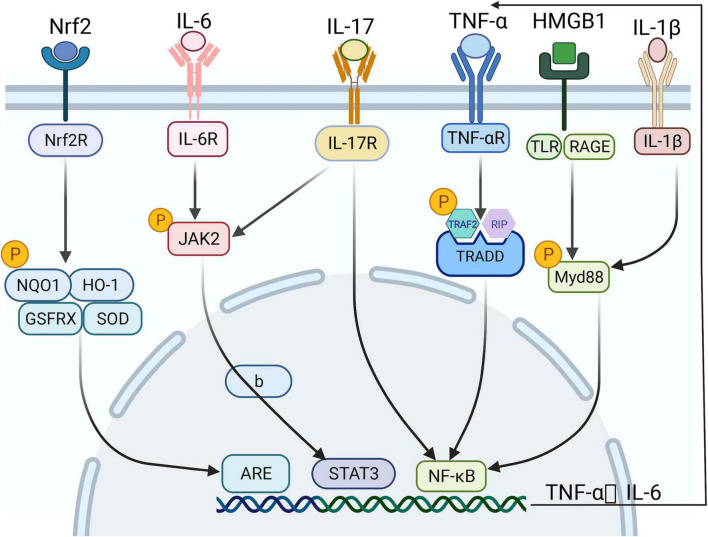
The signaling pathways of lung and intestinal injuries in acute pancreatitis (AP) in different environments and under certain physiological conditions. Several inflammatory activators, such as TNF-α, HMGB1, and proinflammatory factors, activate various intracellular signaling pathways – particularly NF-κB, STAT3, and ARE – by binding specific receptors, thereby releasing a range of inflammatory mediators and promoting each other, forming a vicious cycle.

Inflammatory response plays a key role and has a major effect on the outcome of lung injury in AP (35). Pro-inflammatory factors play a key role in lung injury (36). Pro-inflammatory cytokines are considered to initiate and maintain lung injury (37), and they mainly influence human lung microvascular endothelial cells (HPMECs). Their effect leads to increased alveolar permeability, alveolar tissue fluid extravasation, pulmonary edema, and declined blood oxygen saturation (38). TNF-α, IL-17A, IL-6, IL-1β, and HMGB1 are some of the most important pro-inflammatory cytokines in AP-ALI (Figures 1, 2). The possible cytokines of lung injury in AP are shown in Table 2.

**TABLE 2:** The cytokines of lung and intestinal injuries in AP.

Cytokines	Model	Influence	Function	References
TNF-a	*In vivo*	Upregulation	Promotes the production of inflammatory factors by T cells	(39, 40)
IL-17A	*In vivo*	Upregulation	Enables the body’s immune response	(41, 42)
IL-8	*In vivo*/*In vitro*	Upregulation	Chemotaxis and activation of neutrophils	(43)
IL-1β	*In vivo*	Upregulation	Increase inflammatory cytokines	(16, 34, 44)
HMGB1	*In vivo*	Upregulation	Promotes the release of inflammatory mediators	(45–47)
TXA2, PAF, ET-1, PLA2	*In vivo*	Upregulation	Causes vasospasm, leukocyte and platelet aggregation, thrombosis and vascular endothelial cell damage	(48, 49)

TNF-α, which is released by macrophages and activated fixed circulating monocytes in activated fixed tissues, appears early and plays an important role in the occurrence and development of lung injury in AP (50). It first binds to TNF receptor 1 (TNF-R1) and interacts with adaptor proteins, such as TNF-R1-related death domain (TRADD) protein, TNF-R-related factor 2 (TRAF2), and receptor-interacting protein (RIP). Then, it docks and binds to the mentioned proteins, triggers the intracellular cascade reaction; and finally activates NF-κB (51, 52). NF-κB activation can enhance the transcription of TNF-α genes, thus, creating a vicious feedback loop that amplifies early inflammatory signals and exacerbates the initial inflammatory effects (53) (Figure 1).

IL-17A is closely associated with the pathogenesis of lung injury in AP, and it is mainly expressed by adaptive immune cells populations (39, 54). Study have shown that the expression level of IL-17A is elevated in serum and bronchoalveolar lavage fluid (BALF) in AP-related lung injury (55). Although IL-17A causes lung damage through neutrophil aggregation, which directly leads to pulmonary and pancreatic edema (40), it also acts through synergy with other inflammatory factors, including ligands for TNF-α, IL-1β, and TLRs, and can induce the production of other pro-inflammatory cytokines and chemokines (56). Studies have demonstrated that IL-17A stimulates IL-8 production through synergy with TNF-α and induces an accelerated secretion of IL-6 from human peripancreatic myofibroblasts, which can increase the production of CXCL family of chemokines (neutrophil recruitment) in alveolar type II epithelial cells, and exacerbate airway inflammation and lung ischemia-reperfusion injury (42, 57).

IL-6 is mostly produced by monocytes, macrophages, and dendritic cells (58). It is a multifunctional cytokine with clear pro-inflammatory and anti-inflammatory properties that inhibit proliferation and promote apoptosis and lung injury by activating the JAK2/STAT3 signaling pathway (59, 60). In the early stages of pancreatic lung injury, IL-6 is rapidly synthesized and plays a protective role in host defense. As the injury persists and leukocyte IL-6 production by leukocytes continues to increase, extravasation of alveolar tissue fluid and pulmonary edema occur, leading to impaired lung function, progression of AP, and increased mortality (61).

IL-1β is a member of the IL1 cytokine family. It is produced by activated macrophage preproteins and is hydrolyzed by cystathionine 1 (CASP1/ICE) protein. This cytokine is an important mediator of inflammatory response, and it is involved in various cellular activities, including cell proliferation, differentiation, and apoptosis (62). In lung injury, it is mainly produced upon activation of the NF-κB and JAK2/STAT3 signaling pathways, which intensifies the acute injury process by activating the aggregation of monocytes and macrophages (63, 64). It is expressed only on the surface of activated vascular endothelial cells and initiates the adhesion of leukocytes to vascular endothelial cells, playing an important role in the early development of lung injury in AP (65, 66).

High-mobility group box protein 1 has been proposed as a potent inflammatory mediator in ALI, and the blockade of HMGB1 has led to a significant reduction in lung inflammatory reaction. Luan et al. (34) and Ding et al. (67) demonstrated that the downregulation of HMGB1 can inhibit the activity of NF-κB, inhibit the expression of TNF-α, IL-1β, ICAM-1, and matrix metalloproteinase (MMP) 9 in the lung tissue, and tissue, and therefore decrease the severity of AP-associated ALI. Qu et al. (68) indicated that HMGB1 binds to certain receptors, such as receptor for advanced glycation end products (RAGE) and TLR4, activates the ROS and phosphatidylinositol-3 kinase (PI3K) pathways and myeloid differentiating factor 88 (MyD88), and results in the release of TNF-α, IL-1β, IL-6, and other cytokines contributing to ALI, suggesting that blocking HMGB1 may provide an effective treatment strategy for AP-associated ALI.

### Oxidative Stress in Lung Injury in Acute Pancreatitis

Oxidative stress plays a crucial role in the development of lung injury in AP. An excessive amount of ROS can be generated by activating neutrophils, leading to severe oxidative stress damage (44, 69). Oxidative stress damage not only plays a role in the local damage of the pancreas but also plays a dominant role in damage to other organs, accelerating the occurrence and development of the SIRS and MODS (70, 71). Nuclear factor erythroid 2-related factor 2 (Nrf2) is a redox-sensitive transcription factor of the alkaline locking chain and a major regulator of resistance to oxidative stress, and thus the Nrf2/antioxidant response element (ARE) pathway is the most important endogenous antioxidant signaling pathway in the body (72). Nrf2 can upregulate antioxidant genes and exert antioxidant effects by binding to the promoter sequence, ARE, and activation of Nrf2 can reduce neutrophilic airway inflammation by the upregulation of antioxidants and downregulation of inflammatory cytokines in the airways (73, 74). In AP, Nrf2 dysfunction and function abnormalities of key enzymes in the Nrf2/ARE pathway, such as heme oxygenase-1 (HO-1), quinone oxidoreductase-1 (NQO1), glutathione peroxidase (GSH-Px) and superoxide dismutase (SOD) occur, resulting in loss of antioxidant function and consequent lung tissue damage (75).

### Endocrine Disorders Involving Matrix Metalloproteinases in Lung Injury in Acute Pancreatitis

HPMEs are damaged by inflammatory factors and inflammatory mediators of lung injury in AP (76). HPMECs can secrete MMPs, which are a type of zinc-dependent endopeptidases (77). MMP-2 and MMP-9 are the most extensively studied MPPs. They degrade extracellular basement membrane collagen and alter lung permeability, exacerbating lung injury (78, 79). MMP-2 and MMP-9 have been found to play an important role in ALI, acute respiratory distress syndrome (ARDS), and pulmonary fibrosis (80–82). MMP-9 is mainly involved in the ALI phase, whereas MMP-2 is mainly involved in the subsequent repair and fibrosis phase (83). MMP-9 levels in BALF have been reported to increase in patients with ARDS secondary to septic shock compared to normal controls, suggesting that MMP-9 plays an important role in the development of lung injury in AP (84).

### Other Mechanisms of Lung Injury in Acute Pancreatitis

Abnormalities in the lysosomal degradation pathway, which underlie the emergence of differentiation disorders, can cause impairments in cell development, homeostasis, and survival (85). Studies have shown that abnormalities in the lysosomal degradation pathway, represented by impaired autophagy, play an important role in the evolution of lung injury in AP (86–88). Pulmonary intravascular macrophages (PIMs) damage lung endothelial cells, and they may play a role in another pathogenetic pathway of lung injury in AP (89). Water channel proteins (aquaporins) are a family of membrane proteins involved in the selective transport of water across cell membranes and play a key role in the development of acute pulmonary edema (47). Additional mechanisms, such as activation of ICAM-1 and alteration of circulating protease and phospholipase concentration, have been associated with AP-induced remote lung injury (90). Studies have indicated that ICAM-1 and E-selectin as important inflammatory cytokines are involved in the pathogenesis of AP-associated lung injury, which lead to increased leukocytic infiltration, permeability, proliferation, migration, pulmonary microcirculatory dysfunction and acute respiratory distress syndrome (91, 92).

### The Experimental Animal Models of Acute Pancreatitis-Associated Acute Lung Injury

The experimental animal models of AP and AP-associated ALI have been relatively well-established. Sodium taurocholate, caerulein, or L-arginine are primarily used to create the models (Table 3).

**TABLE 3:** The experimental animal models of pancreatitis-associated ALI.

Models induced	Model	Function	References
Sodium taurocholate	Rat	Emodin protects against acute pancreatitis associated lung injury by Inhibiting NLPR3 inflammasome activation via Nrf2/HO-1 signaling	(93)
Deoxycholic acid sodium salt	Rat	The ICAM-1-mediated JAK2/STAT3 signaling cascade was able to enhance inflammatory responses	(94)
Caerulein	Rat	Inhibition of MMP-9 activity with doxycycline reduced pancreatitis-associated lung injury	(95)
Caerulein and LPS	Mouse	Activation of Nrf2/ARE may be a promising therapeutic target	(74)
L-arginine	Mouse	Reduces acute lung injury by reducing the infiltration of inflammatory cells and inhibiting inflammatory cytokine secretion and cell apoptosis by inhibiting the activation of JAK2-STAT3 signaling	(96)
			

Where rats are were starved overnight and anesthetized with ketamine 40 mg/kg intraperitoneally. The hepatic portal of the bile duct is clamped, and 3.5% sodium taurocholate in a volume of 1 mL/kg is retrogradely injected into the biliopancreatic conduit at a steady pace (0.1 mL/min) (93). The animal models caused by caerulein and LPS have been made. Mice are treated intraperitoneally (i.p.) with 50 μg/kg body weight caerulein (10 mL/kg of body weight) twice every hour (seven injections in total) and 10 mg/kg of lipopolysaccharide (LPS) (10 mL/kg of body weight, i.p.) following the last dosage of caerulein immediately (74). The AP-associated ALI rat model has been established by administering two intraperitoneal injections (1 h apart) of L-arginine at a dose of 4 g/kg body weight (96).

## Mechanisms of Intestinal Injury in Acute Pancreatitis

**FIGURE 3:**
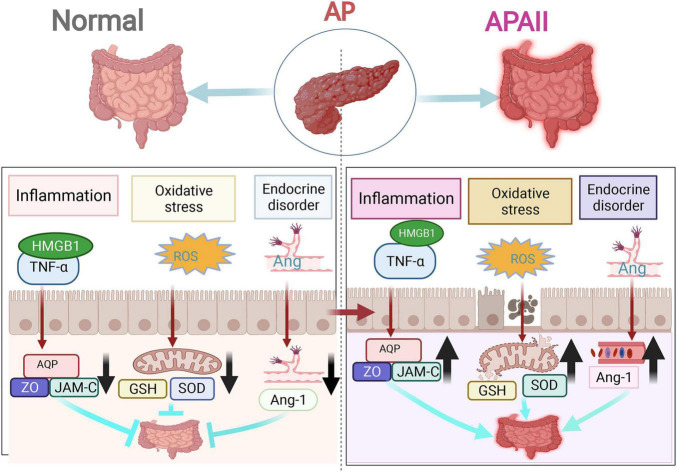
The three main mechanisms of intestinal injury in acute pancreatitis (AP). The three most highlighted mechanisms of intestinal injury in AP are inflammatory response outbreak, oxidative stress damage, and endocrine disorders.

In intestinal injury caused by AP, the intestinal barrier becomes damaged the earliest. The pathological changes are closely related to the destruction of the intestinal mucosal structure and an increase in intestinal mucosal permeability (97, 98). The effects of intestinal barrier failure in AP manifest as intestinal immune deficiency, intestinal microflora disturbance, increased intestinal permeability, and excessive release of inflammatory mediators (like endotoxin, cytokines, chemokines, DAMPs, and mRNAs) (16, 97). Based on the relevant literature, this review summarizes the mechanisms of intestinal injury, including inflammatory response burst, oxidative stress injury, and endocrine disruption represented by angiopoietin (Ang) and mitochondrial injury (Figure 3).

### Inflammatory Response to Intestinal Injury in Acute Pancreatitis

The inflammatory response plays a major role in the development and prognosis of intestinal injury in AP (16). Inflammatory cells and factors accumulate and infiltrate during the development of AP, leading to the disruption of the intestinal barrier and intestinal mucosal structure and an increase in intestinal mucosal permeability. The disruption of the intestinal barrier function is mainly manifested in two aspects: intestinal mucosal epithelial barrier dysfunction (mechanical, chemical, immunological, and biological barrier dysfunction) and intestinal capillary endothelial barrier dysfunction (99). In contrast, most of the inflammatory responses are caused by mechanical and chemical barrier dysfunction.

The intestinal mechanical barrier comprises intestinal epithelial cells (IECs) and tight intercellular junctions. IECs separate the intestinal lumen from the lamina propria. The tight junctions are the main determinants of paracellular permeability (100). IECs ensure proper digestion and barrier function with a high turnover rate of 4–5 days (101). Uncontrolled inflammatory response of the intestinal epithelium is a characteristic feature of intestinal barrier failure in severe AP. The caspase-3 pathway can be stimulated by an inflammatory factor, TNF-α, leading to severe apoptosis of intestinal mucosal cells and destruction of intestinal mucosal structures (102). Another inflammatory factor, HMGB1, activates RAGE and TLRs, which, in turn, activate the NF-κB signaling, release inflammatory mediators, and enhance the binding of ARE elements to downstream target mRNAs (such as those for TNF-α, IL-6, and IL-8), and maintain the stability of target mRNAs. As a result, they increase the translation of related proteins and induce a strong pro-inflammatory effect (16).

The intestinal chemical barrier consists of mucin (MUC), antimicrobial peptides (AMPs), and other digestive enzymes. AMPs have sterilizing and anti-inflammatory functions. Additionally, they promote tissue repair. In the intestine, AMPs, especially β-defensins, in the intestine are reduced due to the aggregation and infiltration of inflammatory cells in AP, increasing the rate of intestinal bacterial translocation and the possibility of retrograde infection (103). MUC2, one of the most common forms present of MUC, is the main component of the intestinal chemical barrier covering IECs and forms the intestinal mucus layer. It is the first line of defense of the intestinal mucosal barrier; therefore, it is the most vulnerable to inflammatory reactions and loss of barrier function (104) (Table 2).

The intestinal capillary endothelial barrier is a semi-selective barrier composed of a single layer of endothelial cells surrounding the vascular lumen and basement membrane. The occurrence of intestinal capillary endothelial barrier impairs capillary permeability, which affects intestinal mucosal permeability and causes intestinal edema. Capillary permeability is determined by three factors: endothelial cells, inter-endothelial connections, and basement membrane (49). Studies have shown that aquaporin 1 (AQP-1), MMP-9, and junctional adhesion molecule-C (JAM-C) play key roles in the regulation of capillary permeability in AP (105). Under the stimulation of inflammatory factors, intestinal permeability can increase when AQP-1 is downregulated, leading to an imbalance in water transfer and homeostasis between the cells and blood vessels. Microvascular permeability can increase when MMP-9 is decreased, leading to damage to the basement membrane. JAM-C is localized at cell junctions, increasing intercellular permeability when affected, which, in turn, increases intestinal mucosal permeability and causes intestinal edema (95).

### Oxidative Stress of Intestinal Injury in Acute Pancreatitis

During oxidative stress, an imbalance between oxidative and antioxidant action occurs in the body. Inflammatory infiltration of neutrophils, increased secretion of proteases, and production of oxidative intermediates, such as oxygen radicals and lipid peroxides, lead to IEC damage in the early stages of AP (106). Oxygen radicals and lipid peroxides can be produced in damaged intestinal tissues by activated immune cells and alveolar cells, which can activate the Nrf2/ARE antioxidant signaling pathway (79), resulting in a decrease in total GSH and SOD levels (107, 108). As a result, intestinal barrier function, intestinal mucosal structure, and intestinal mucosal permeability become disrupted (109).

### Endocrine Disorders of Intestinal Injury in Acute Pancreatitis

Ang has an important role in the pathology of intestinal capillary leakage syndrome in SAP. Normally, Ang-1 not only promotes endothelial cell chemotaxis and aggregation but also inhibits apoptosis, inflammation, exudation, and leukocyte adhesion (110). Additionally, it regulates the proliferation of endothelial cells and vascular smooth muscle cells, and promotes vascular maturation, which is important for maintaining vascular stability and integrity (111). In AP, a significant decrease in serum Ang-1 levels, a significant increase in the number of inflammatory factors in the serum, an increase in capillary permeability of the tissues, an increase in alveolar fluid, and the appearance of pulmonary edema are all observed. Related studies have shown that Ang1 not only effectively alleviated intestinal capillary leakage in a rat model of AP but also played a positive role in the protection of intestinal microcirculation. Additionally, it significantly upregulated the expression of AQP-1 and downregulated the expression of MMP-9 and JAM-C protein to provide intestinal protection (49).

### Other Mechanisms of Intestinal Injury in Acute Pancreatitis

Mitochondria are central to cellular viability and function, controlling control many physiological metabolic processes (112, 113). Disorders of ROS and cytochrome C metabolism, which occur in mitochondria, can lead to abnormalities in intestinal metabolism (114). Significant mitochondrial dysfunction occurs in the jejunum in the early stages of severe AP in rat models (115). Additionally, swelling of mitochondria in the colonic mucosal epithelium occurs. In edematous pancreatitis, degeneration and rupture of colonic mitochondria are present in necrotizing pancreatitis, suggesting that the damage to the mitochondria is closely related to intestinal injury in AP (116).

## Common Mechanisms of Lung and Intestinal Injuries in Acute Pancreatitis

The lungs and intestine are organs that are directly damaged organs in AP. Mesenteric lymph (ML) is the direct axial connection between the intestine and lungs (117). Physiologically, the lungs and large intestine can interact with each other to promote homeostasis, whereas pathologically, the balance and interaction between the two axes are disturbed. Lung-gut syndrome is a condition in which the lungs and large intestine become affected concurrently; respiratory and digestive tract disorders occur together (118). This interaction is consistent with the theory of *the lung being connected with the large intestine* in Chinese medicine. Inflammatory mediators reaching the lung and intestinal tissues cause an inflammatory response, oxidative stress, and endocrine hormone secretion disorders, impairing the balance axis between the lung and intestine.

### Inflammatory Response in the Imbalance of the Lung-Gut Axis

Acute pancreatitis is an acute necrotic and inflammatory process that suddenly occurring around and inside the pancreas (119). Overactivation of the inflammatory response is important in the course of pancreatitis, including the production and activation of inflammatory factors and the accumulation of pro-inflammatory mediators. The main inflammatory factors, including TNF-α, IL-17, IL-1, and IL-8, are released into the bloodstream. They accumulate in the lungs and intestine, activating the NF-κB signaling pathway, amplifying the inflammatory effect, and inducing a systemic inflammatory response. These processes lead to inflammatory injury in the lungs and intestine (120). Thromboxane A2 (TXA2), platelet-activating factor (PAF), endothelin 1 (ET-1), phospholipase A2 (PLA2), and IL-1β can cause vasospasm, white blood cell and platelet aggregation, thrombosis, and vascular endothelial cell damage, leading to the loss of the intestinal barrier, which in turn causes lung injury (121). The main pro-inflammatory mediators include HMGB1 and TLR-4. HMGB1 is released by necrotic acinar cells and acts on its receptors, ARGE and TLR (122). Chen et al. (123) found that the expression of HMGB1 is positively related to intestinal barrier failure and inhibition of HMGB1 can significantly improve intestinal injury. Furthermore, HMGB1 activates the NF-κB signaling pathway and releases several downstream inflammatory factors by myd88 and appears during an early inflammatory response (124). Relevant studies have shown that the levels of HMGB1 and TLR-4 can be used as important indicators of AP progress (125) (Figure 2 and Table 2).

### Oxidative Stress in the Imbalance of the Lung-Gut Axis

During AP, Ca^2+^ overload, inflammatory mediators, and zymogen activation can damage the arterial muscles and vascular endothelial cells in the pancreatic lobules, resulting in pancreatic vasoconstriction, shunting, and insufficient perfusion. Intestinal mucosal tissue is the most susceptible to insufficient perfusion (126). When the intestinal mucosa is signaled by ischemia and hypoxia, xanthine oxidase and hypoxanthine accumulate in the intestinal tissue, leading to adenosine triphosphate (ATP) depletion due to insufficient oxidative phosphorylation. In the subsequent reperfusion, the body converts hypoxanthine to xanthine, and then releases superoxide ions to generate more free oxygen radicals. The Nrf2/ARE antioxidant signal pathway is activated, causing lipid peroxidation and cell membrane damage, which accelerate intestinal barrier loss and lung function impairment (127). Liu et al. (43) have found that SAP can change the hemodynamics of rats, leading to severe microcirculation disturbances in the pancreas and other organs (especially the lungs) and causing redox imbalance. These changes result in hyperosmotic edema of the lungs and increased alveolar fluid level (10, 15), as well as other pathological changes, including the destruction of intestinal mucosal structure and changes in intestinal mucosal permeability (35) (Figure 2).

### Endocrine Disorders in the Imbalance of the Lung-Gut Axis

The intestine is not simply a digestive organ: it has certain endocrine functions. The endocrine hormones secreted by the intestine play important roles in gastric content transport and digestion. Hormones, such as secreted cholecystokinin octapeptide (CCK-8) and blood vessel active intestinal peptide (VIP), can cause lung and intestinal damage in AP under pathological conditions (48). Blood CCK is mainly derived from intestinal secretory endothelial cells. Related studies have shown that it not only promotes gall bladder contraction but also relaxes the sphincter of Oddi and protects the gastric mucosa. Additionally, it can relax tracheal muscles, activate pulmonary interstitial macrophages (PIM), and reduce the occurrence of endotoxemia-related pneumonia. Although CCK is derived from enteroendocrine endothelial cells, it can cause lung and intestinal damage concurrently. VIP is a linear peptide widely distributed in the gastrointestinal tract and lungs. Its function is mainly to promote glandular secretion. It is negatively correlated with gastrointestinal motility. It can dilate systemic and pulmonary blood vessels, and inhibit the proliferation of pulmonary artery smooth muscle cells (128). Animal experiments have proven that VIP can reduce pulmonary hypertension by reducing pulmonary vascular resistance (45).

### Other Mechanisms in the Imbalance of the Lung-Gut Axis

Water channel proteins play a vital role in maintaining the water homeostasis of the lungs and intestine. AQPs are a type of membrane water channels, consisting of six transmembrane spiral segments and two shorter spiral segments that do not span the entire membrane. Their main function is to promote water transport in passive transport, and AQPs are widely distributed in mammalian organs (46). Currently, 13 AQPs have been discovered, and at least eight AQPs have been shown to transport water in humans and rodents (47). It was previously shown that the expression levels of AQP-1 and AQP-5 were decreased in patients with pulmonary edema after a viral infection were decreased (129). Sakai et al. (130) that the genes of *AQP4* and *AQP8* are mainly expressed in the mouse colon. The regulation of transepithelial fluid transport in the gastrointestinal tract is based on ion and water transport by AQPs (131). Additionally, the appearance of pulmonary and bowel dysfunction is closely related to intra-abdominal hypertension (IAH) and/or abdominal cavity syndrome (ACS). Reduced IAH maintains the integrity of the intestinal barrier, promotes the recovery of intestinal function, inhibits inflammation, and increases blood oxygen saturation (SpO_2_) (132). To reduce tissue damage of the intestines and lungs (133), thus, other mechanisms of lung and bowel damage in AP can be targeted to reduce damage to the intestine and lungs (133).

## Potential Targets for the Protection of Lung and Intestinal Injuries in Acute Pancreatitis

Studies have shown that lipoxin A4 inhibits the activation of TNF-α/TNF-R1 and downstream signals, such as TRADD, TRAF2, and RIP. The inhibition influences the activity of the NF-κB pathway, and consequently inhibits the production of inflammatory mediators and the impairment of pulmonary intestinal injury (94, 134). According to another study, TLR4, which is a key receptor for the recognition of pathogen-associated molecular patterns (PAMPs), may provide valuable guidance for the treatment of pulmonary intestinal injury in SAP (135).

BML-111, which is a commercially synthesized lipoxin receptor agonist, protects against emotion-induced LAI in mice by modulating the Nrf2/ARE signaling pathway (74). Isoglycyrrhizinate alleviates AP in mice by inhibiting oxidative stress and modulating the Nrf2/HO1 pathway (31). The protective effect of tanshinone IIA in AP in mice occurs by inhibiting oxidative stress through the Nrf2/ROS pathway (136). Protective effects of rhodopsin against AP-induced lung injury occur by the inhibition of NLRP3 inflammasome activation specifically activated by the Nrf2/HO-1 pathway (93). HMGB1 inhibitors reduce the severity of SAP by reducing intestinal permeability through the reduction in claudin-2 and occludin expression (25). Moreover, hydrostatin-SN10 significantly reduces IL-6 levels, reduces inflammatory response, and improves lung tissue injury by inhibiting JAK2/STAT3 phosphorylation (137).

## Conclusion and Future Perspectives

This review described the three common mechanisms of lung and intestinal injury in AP. The common mechanisms of lung and intestinal injury in AP are inflammatory response, oxidative stress, and endocrine hormone secretion disorder. However, few studies have reported concurrent treatment options for lung and intestinal injury in AP. Therefore, future studies are required to investigate common treatment options for lung and intestinal injury in AP. The mechanism of inflammatory response is the most important element in lung and intestinal injury, because it is generally considered to be effective in the common prevention and treatment of lung and intestinal injury in AP. However, details regarding the involved pro-inflammatory mediators and cells are still lacking.

Mesenteric lymph can concurrently cause lung and intestinal injury in AP. Therefore, surgical diversion of ML represents a treatment option. An inflammatory response is an unclear mechanism of lung and intestinal injury in AP. Therefore, currently, the greatest challenge of studies on lung and intestinal injury in AP is the elucidation of the specific mechanisms and their precise targets. Accurate detection of these mechanisms and targets and demonstration of the remarkable effects of multitarget integration will be useful for lung and intestinal injury in AP in clinical practice. Additionally, immunotherapy is also a promising approach, and it could be an effective strategy targeting and altering the activities of immune cells in AP, which might have long-term therapeutic effects.

## Author Contributions

YW and DL organized thoughts for the manuscript and revised the manuscript. LW drafted the manuscript and gathered information for the manuscript. DY revised the first manuscript. ZW, YH, and MB translated and revised the manuscript. YZ, DY, and BS offered opinions for the drawing of diagrams and charts in the manuscript. All authors read and approved the final version of the manuscript.

## Conflict of Interest

The authors declare that the research was conducted in the absence of any commercial or financial relationships that could be construed as a potential conflict of interest.

## Publisher’s Note

All claims expressed in this article are solely those of the authors and do not necessarily represent those of their affiliated organizations, or those of the publisher, the editors and the reviewers. Any product that may be evaluated in this article, or claim that may be made by its manufacturer, is not guaranteed or endorsed by the publisher.

## Abbreviations

AP: acute pancreatitis
ALI: acute lung injury
DAMPs: damage-associated molecular patterns
HMGB1: high-mobility group box protein 1
ML: mesenteric lymph
MMPs: matrix metalloproteinases
TNF- α: tumor necrosis factor- α
IL-17: interleukin-17
IL-6: interleukin-6
IL-1 β: interleukin-1 β
TNF-R1: tumor necrosis factor-R1
TRADD: tumor necrosis factor receptor 1 related death domain protein
TRAF2: tumor necrosis factor receptor related factor 2
TLR4: Toll-like receptors 4
NLRP3: NOD-like receptor protein 3
STAT3: signal transducer and activator of transcription 3
AP-1: activator protein-1
MAPK: mitogen-activated protein kinases
NF- κ B: nuclear factor kappa B
SIRS: systemic inflammatory response syndrome
MODS: multiple organ dysfunction syndrome
Nrf2: Related factor 2
ARE: antioxidant response element
AQPs: Water channel proteins
Ang: angiopoietin
IEC: intestinal epithelial cells
AMPs: antimicrobial peptides
JAM-C: junctional adhesion molecule-C
GSH: glutathione
SOD: superoxide dismutase
ROS: reactive oxygen species
VIP: blood vessel active intestinal peptide.
